# Bactericidal Effect of Photodynamic Therapy, Alone or in Combination with Mupirocin or Linezolid, on *Staphylococcus aureus*

**DOI:** 10.3389/fmicb.2017.01002

**Published:** 2017-05-31

**Authors:** Vanesa Pérez-Laguna, Luna Pérez-Artiaga, Verónica Lampaya-Pérez, Isabel García-Luque, Sofía Ballesta, Santi Nonell, Manuel P. Paz-Cristobal, Yolanda Gilaberte, Antonio Rezusta

**Affiliations:** ^1^IIS AragónZaragoza, Spain; ^2^Department of Microbiology, Hospital Universitario Miguel ServetZaragoza, Spain; ^3^Department of Microbiology, University of SevillaSeville, Spain; ^4^Institut Químic de Sarrià, Universitat Ramon LlullBarcelona, Spain; ^5^Department of Dermatology, Hospital San JorgeHuesca, Spain; ^6^Department of Microbiology, Preventive Medicine and Public Health, University of ZaragozaZaragoza, Spain

**Keywords:** *S*. *aureus*, antibiotics, rose Bengal, methylene blue, photoinactivation

## Abstract

Antibiotic treatments frequently fail due to the development of antibiotic resistance, underscoring the need for new treatment strategies. Antimicrobial photodynamic therapy (aPDT) could constitute an alternative therapy. In bacterial suspensions of *Staphylococcus aureus*, which is commonly implicated in cutaneous and mucosal infections, we evaluated the *in vitro* efficacy of aPDT, using the photosensitizing agents rose bengal (RB) or methylene blue (MB), alone or combined with the antibiotics mupirocin (MU) or linezolid (LN). RB or MB, at concentrations ranging from 0.03 to 10 μg/ml, were added to *S. aureus* ATCC 29213 suspensions containing >10^8^ cells/ml, in the absence or presence of MU or LN (1 or 10 μg/ml). Suspensions were irradiated with a white metal halide (λ 420–700 nm) or light-emitting diode lamp (λ 515 and λ 625 nm), and the number of viable bacteria quantified by counting colony-forming units (CFU) on blood agar. Addition of either antibiotic had no significant effect on the number of CFU/ml. By contrast, RB-aPDT and MB-aPDT effectively inactivated *S. aureus*, as evidenced by a 6 log_10_ reduction in bacterial growth. In the presence of MU or LN, the same 6 log_10_ reduction was observed in response to aPDT, but was achieved using significantly lower concentrations of the photosensitizers RB or MB. In conclusion, the combination of MU or LN and RB/MB-aPDT appears to exert a synergistic bactericidal effect against *S. aureus in vitro*.

## Introduction

Microbial infections are a leading causes of mortality worldwide, largely due to the development of multidrug resistance (Livermore, 2009). In hospitals, *Staphylococcus aureus*, a Gram-positive bacteria, has become the most commonly isolated pathogen involved in serious diseases (Emori and Gaynes, 1993), and the emergence of methicillin-resistant *S. aureus* (MRSA) strains worldwide poses serious risks to patients with immunological diseases (Orrett and Land, 2006; Boucher et al., 2009). Several antibiotics, including mupirocin (MU) and linezolid (LN), have proven effective against both methicillin-resistant and non-resistant strains of *S. aureus* (Tallón et al., 2002; Larru et al., 2016). MU is one of the most frequently used antibiotics for topical treatment of *S. aureus* skin infections (Saderi et al., 2008), while LN is more commonly administered intravenously (Cattaneo et al., 2013).

Although results vary depending on the studied strain and its geographical localization, several recent studies suggest that the antibiotic resistance of *S. aureus* is on the rise, underscoring the need for new treatment strategies (Orrett, 2008; Saderi et al., 2008; Gu et al., 2013; Gostev et al., 2015; Larru et al., 2016).

Antimicrobial photodynamic therapy (aPDT) is based on the use of photosensitizer molecules that are activated by harmless visible light in the presence of oxygen. This combination generates reactive oxygen species that can oxidize many biological molecules, including proteins, nucleic acids, and lipids, leading to cell death (Henderson and Dougherty, 1992). Given the rapid and selective union that forms between photosensitizers and the cells of microorganisms, aPDT has been proposed as an alternative treatment for localized infections (Dai et al., 2012).

Phenothiazinium derivates and fluorescein-like molecules, such as methylene blue (MB) and rose bengal (RB), respectively, are polycyclic aromatic molecules that have been used as photosensitizers in aPDT, demonstrating the efficacy of this approach in inactivating resistant forms of bacteria that are not easily killed by conventional antibiotics. Initial *in vitro* studies have produced promising results, supporting the use of these compounds in the treatment of microbial infections (Demidova and Hamblin, 2005; Tanaka et al., 2012). The combination aPDT and conventional antibiotics to treat staphylococcal infections has also shown significant potential, opening up new avenues in the quest for novel therapies for these dangerous and recurrent infections (Di Poto et al., 2009; Sbarra et al., 2009).

The aim of this study was to compare the *in vitro* efficacy of aPDT using the photosensitizers RB or MB (RB-aPDT and MB-aPDT), combined with the antibiotics MU or LN, against *S. aureus*.

## Materials and methods

### Chemicals and media

– Solvent: Bidistilled water.
– Culture Media: Columbia blood agar (BA) (Oxoid®; Madrid, Spain).
– Antibiotics: Mupirocin (MU) and linezolid (LN), both from Sigma-Aldrich® (Madrid, Spain). Both antibiotics were applied at concentrations of 1 μg/ml and 10 μg/ml, both of which exceed the minimum inhibitory concentration of the strain (EUCAST) but do not cause significant damage alone in *S. aureus* strains. Respective controls were performed.
– Photosensitizers: Methylene blue (MB), purchased from Sigma-Aldrich® (Madrid, Spain), and (RB), from Sigma-Aldrich-Fluka® (Madrid, Spain). Stock MB and RB solutions were prepared and diluted in bidistilled water immediately prior to use. All solutions were prepared and handled under light-restricted conditions. Concentrations ranged from 0.03 to 10 μg/ml. This concentration range was chosen based on unpublished results from previous experiments performed in our laboratory using 2-fold serial dilutions from 640 to 0.03 μg/ml of both photosensitizers.

### Light sources

Two light-emitting diode (LED) and one white metal halide (WMH) lamps were used.

**Figure 1:**
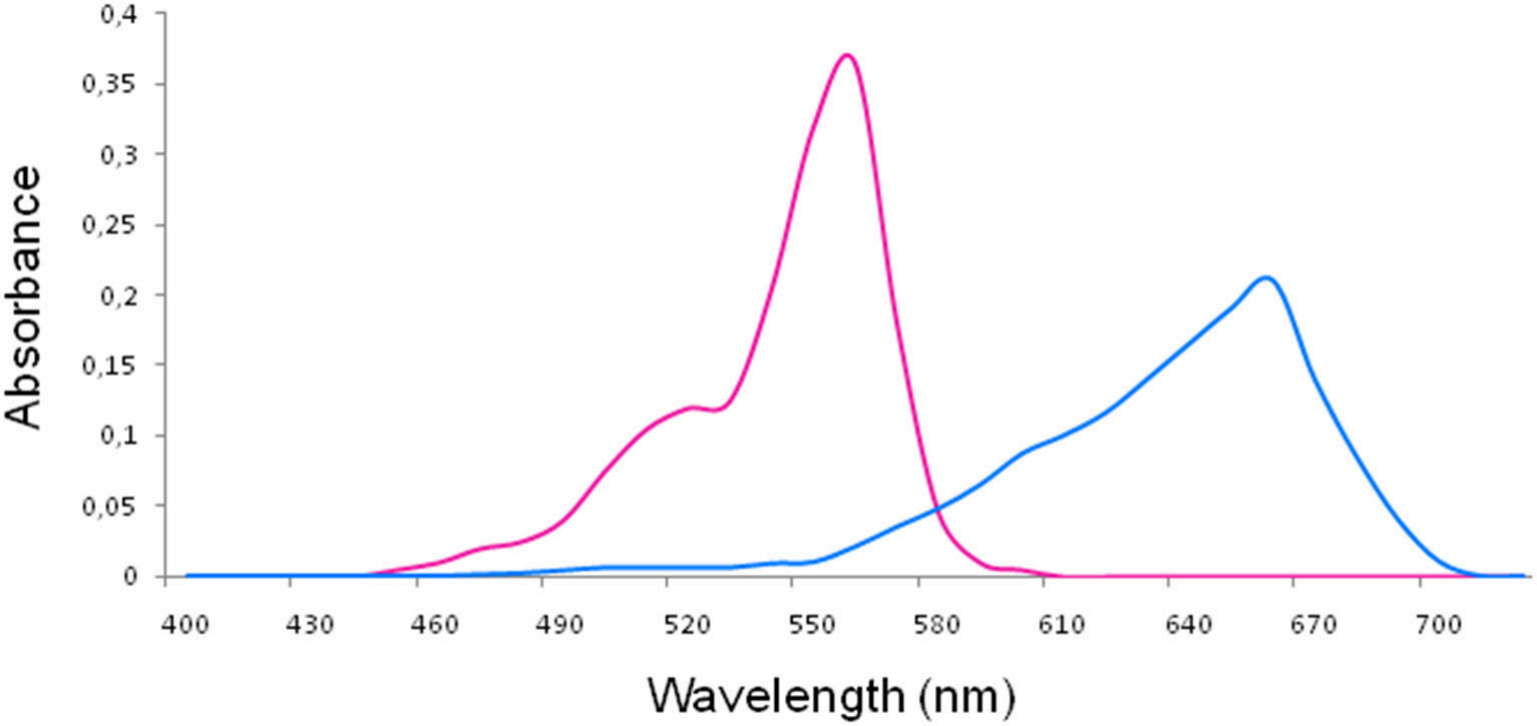
Absorption spectra of rose bengal (left) and methylene blue (right).

**Figure 2:**
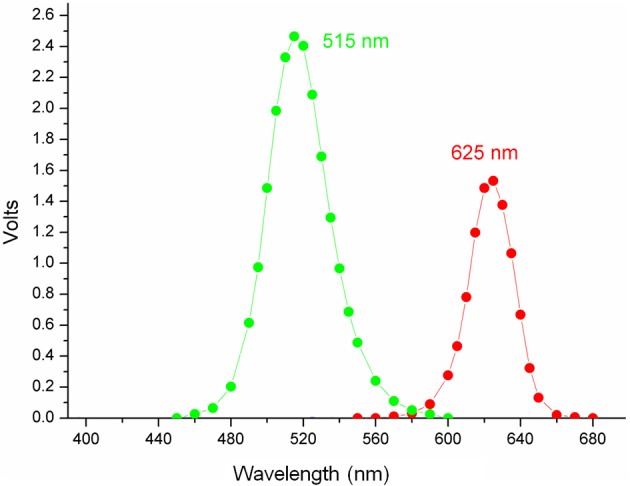
Emission spectra of LED lamps.

For RB (maximum absorption λ, 557 nm) and MB (maximum absorption λ, 665 nm) (Soria-Lozano et al., 2015; Figure 1) aPDT was performed using LED lamps emitting at 515 ± 10 nm (5.8 mW/cm^2^) and 625 nm ± 10 nm (7 mW/cm^2^) (Figure 2), respectively, with fluences of 18 J/cm^2^ and 37 J/cm^2^.

**Figure 3:**
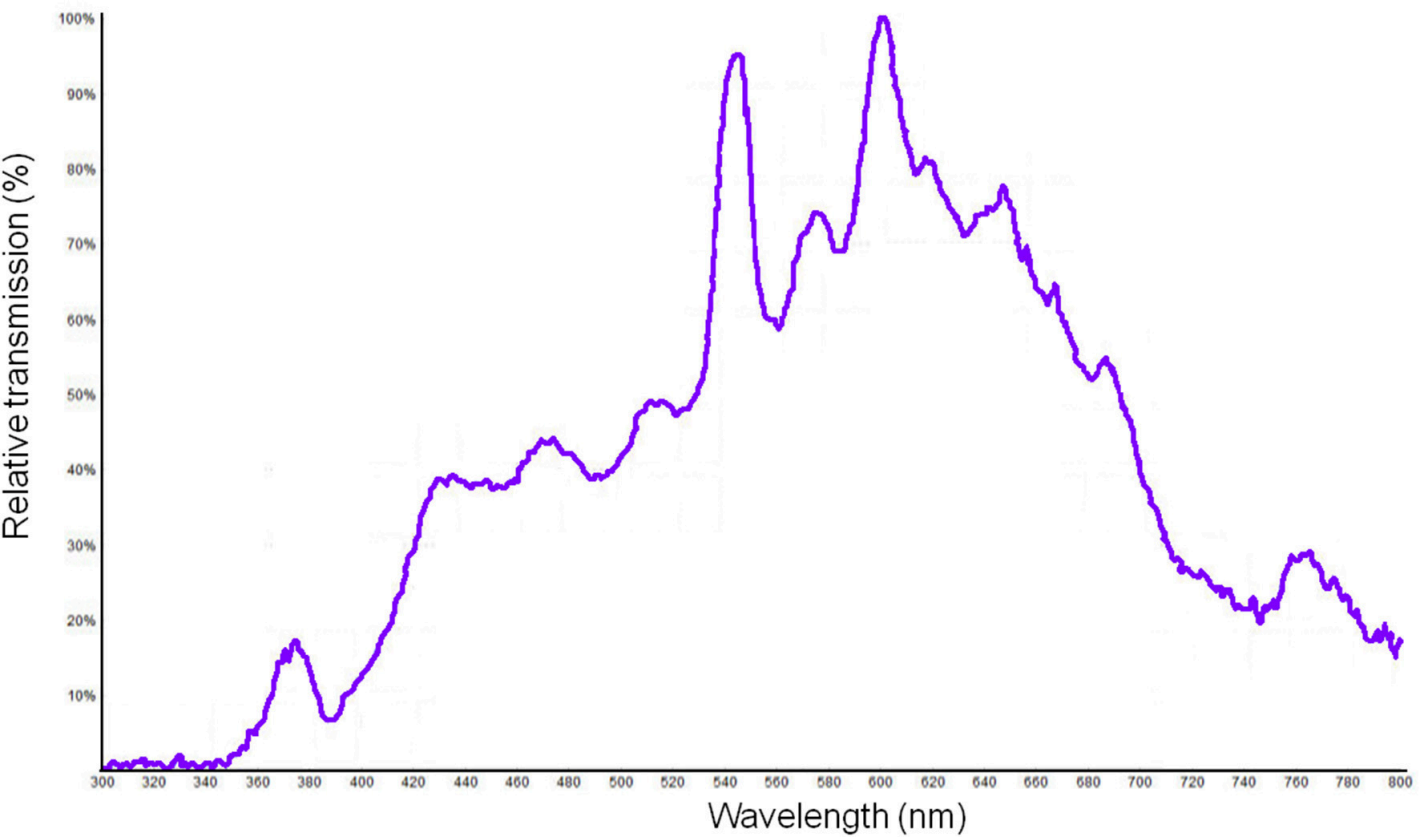
Relative emission curve of the white metal halide (WMH) lamp.

For both photosensitizers a WMH lamp emitting at 420–700 nm (Soria-Lozano et al., 2015) at a fluence of 37 J/cm^2^ was used (Figure 3). The lamp had an irradiance of 90 mW/cm^2^. The specific irradiance values at the maximum absorption λ of RB and MB are 292 μW/cm^2^ at 557 nm and 300 μW/cm^2^ at 665 nm, respectively.

### Microorganisms and growth conditions

*Staphylococcus aureus* ATCC 29213 was acquired from the American Type Culture Collection (ATCC, Rockville, MD, USA). Microorganisms were grown aerobically overnight on BA plates at 35°C. The inoculum was prepared in bidistilled water and adjusted to 0.5 ± 0.03 on the McFarland scale [concentrations in the range of >10^8^ colony-forming units per ml (CFU/ml)]. Cell viability was assessed in serial dilutions of suspension controls by counting CFU after incubation overnight at 35°C on BA. For aPDT assays, samples were grown on BA in the same conditions as controls.

### *In vitro* photodynamic treatment of bacteria

Bacteria seeded on BA were cultured overnight at 35°C, and suspensions of the desired McFarland value (>10^7^ CFU/ml) were prepared in bidistilled water and deposited into 96-well microtiter plates. Varying concentrations of the photosensitizer (RB or MB; concentration range 0.03–10 μg/ml) were added, in the presence or absence of MU or LN (1 μg/ml or 10 μg/ml). The final volume of each well was 100 μl. Irradiation proceeded with no preincubation period; the suspensions were immediately subjected to irradiation with fluences of either 18 J/cm^2^ or 37 J/cm^2^ using LED lamps and 37 J/cm^2^ using the WMH lamp. Control samples were subjected to identical treatment, in the absence or presence of the photosensitizer, and were either kept in darkness or irradiated to evaluate the effect of each parameter. After completing the aPDT protocol, samples and controls were cultured on BA and incubated overnight at 35°C. The effectiveness of aPDT treatment was assessed by counting the number of CFU/ml using a Flash and Go automatic colony counter (IUL, S.A, Spain) and comparing the results with controls. All experiments were carried out at least 5 times. A reduction the number of CFU/ml of 6 log_10_ was considered indicative of bactericidal activity.

## Results

### Photoinactivation of bacteria by RB-aPDT or MB-aPDT

**Figure 4:**
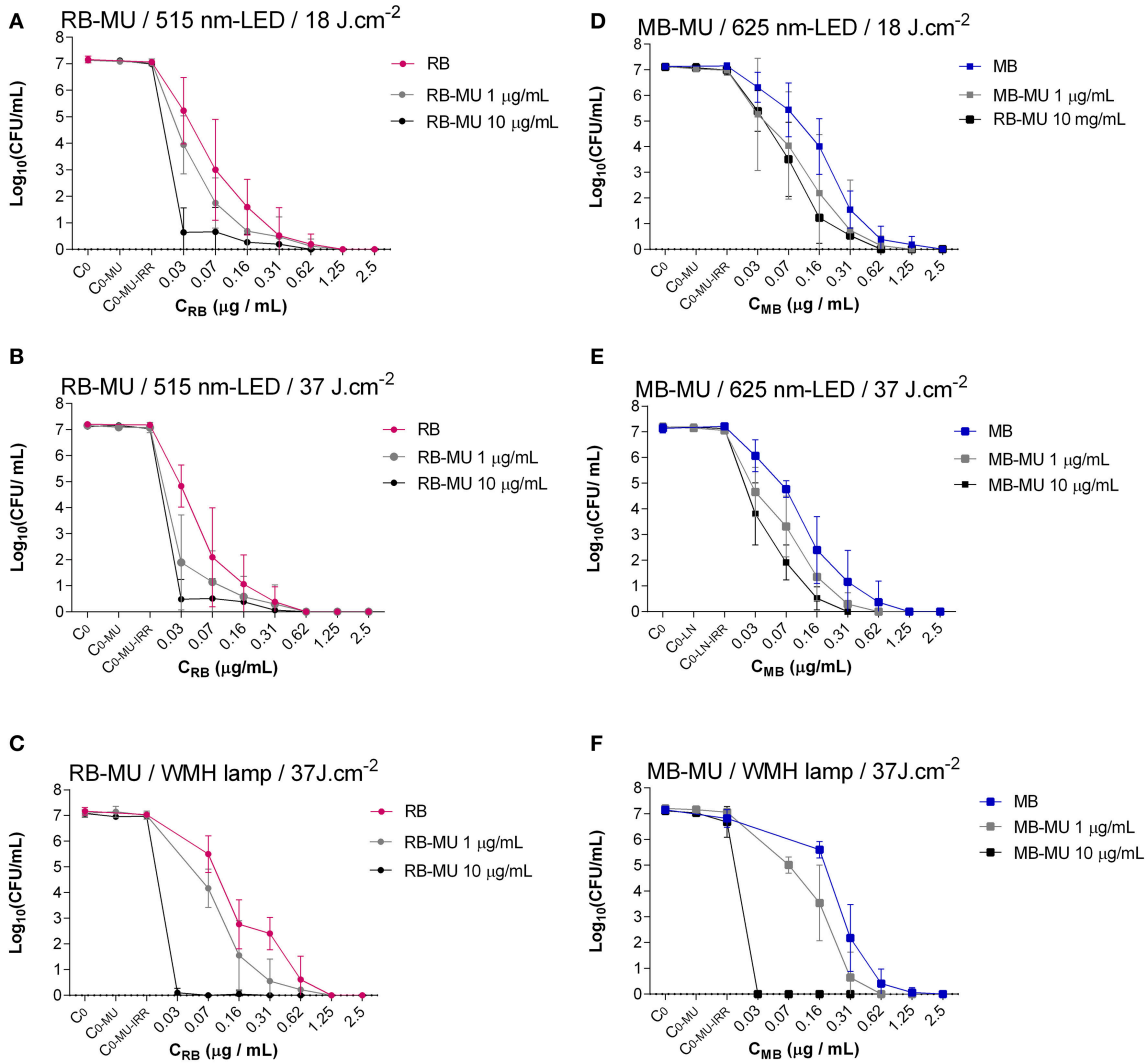
Photoinactivation of *S. aureus* using different concentrations of RB (left) or MB (right) combined with MU treatment. **(A,D)**, Constant fluence of 18 J/cm^2^ with LED lamp; **(B,E)**, constant fluence of 37 J/cm^2^ with LED lamp; **(C,F)**, constant fluence of 37 J/cm^2^ with WMH lamp. C_0_, Control of inoculum (Without photosensitizer, without antibiotic, without irradiation); C_0−MU_, Control of antibiotic (Without photosensitizer, with antibiotic, without irradiation); C_0−MU−IRR_, Control of irradiation -added to the effect of antibiotic-(Without photosensitizer, with antibiotic, with irradiation).

**Figure 5:**
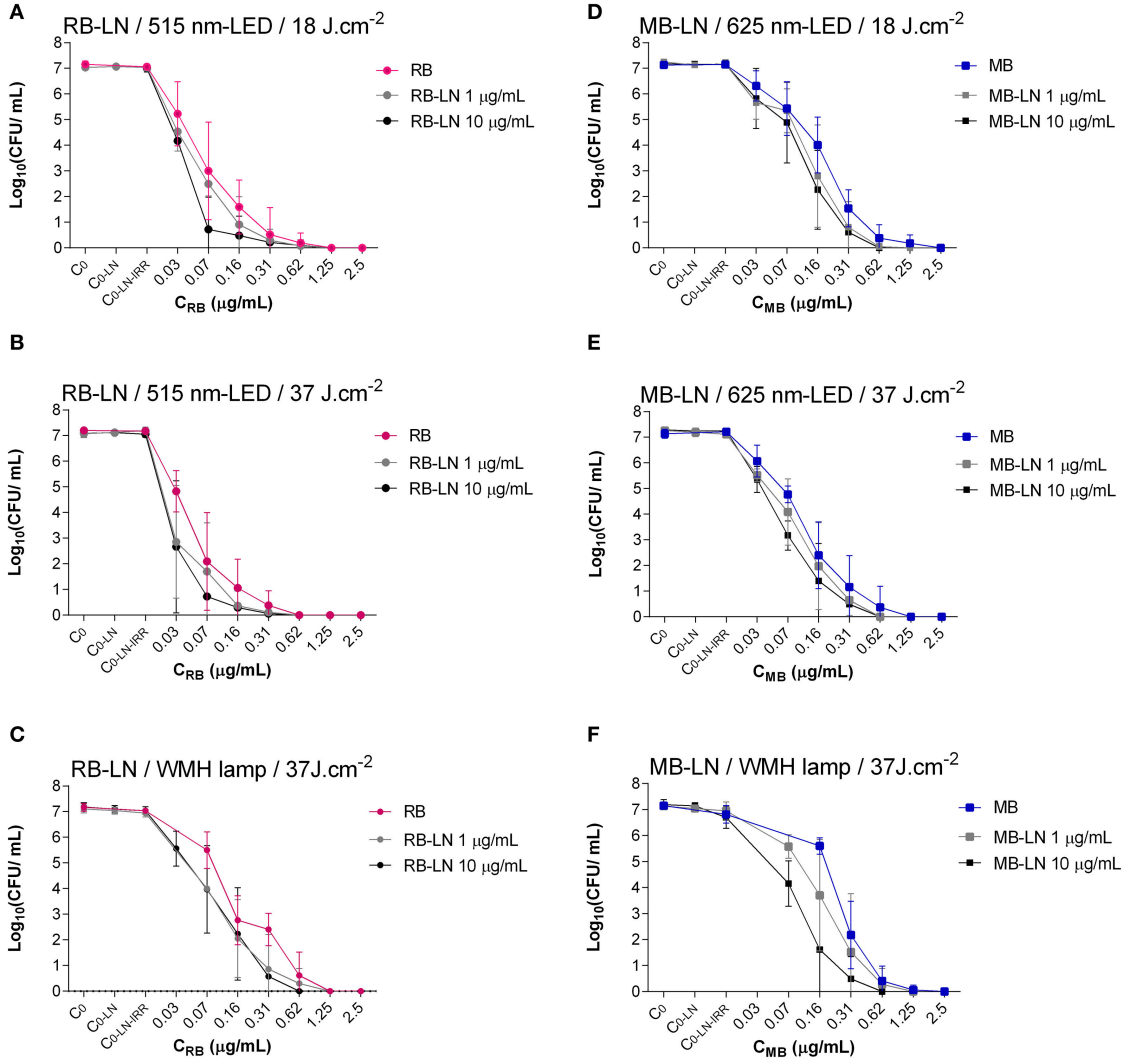
Photoinactivation of *S. aureus* with different concentrations of RB (left) or MB (right) combined with LN treatment. **(A,D)** Constant fluence of 18 J/cm^2^ with LED lamp; **(B,E)**, constant fluence of 37 J/cm^2^ with LED lamp; **(C,F)**, constant fluence of 37 J/cm^2^ with WMH lamp. C_0_, Control of inoculum (Without photosensitizer, without antibiotic, without irradiation); C_0−LN_, Control of antibiotic (Without photosensitizer, with antibiotic, without irradiation); C_0−LN−IRR_, Control of irradiation -added to the effect of antibiotic-(Without photosensitizer, with antibiotic, with irradiation).

aPDT effectively inactivated *S. aureus* ATCC 29213, resulting in 6 log_10_ reduction in bacterial growth in all assays (Table 1, Figures 4, 5).

**Table 1:** Range of minimum photosensitizer concentrations (μg/ml) required to reduce *S. aureus* growth by 6 log_10_.

	**MB 625 nm-LED-lamp**		**MB WMH-lamp**	**RB WMH-lamp**	**RB 515 nm- LED-lamp**	
	**Fluence 18 J/cm^2^**	**Fluence 37 J/cm^2^**	**Fluence 37 J/cm^2^**	**Fluence 37 J/cm^2^**	**Fluence 18 J/cm^2^**	**Fluence 37 J/cm^2^**
PS	0.62	0.62	0.62	0.62	0.31	0.31
PS+LN 1 μg/ml	0.31	0.31	0.62	0.31	0.16	0.16
PS+LN 10 μg/ml	0.31	0.31	0.31	0.31	0.07	0.07
PS+MU 1 μg/ml	0.31	0.31	0.31	0.31	0.16	0.16
PS+MU 10 μg/ml	0.31	0.16	≤ 0.03	≤ 0.03	0.03	0.03

*LED, Light-emitting diode; LN, linezolid; MB, methylene blue; MU, mupirocin; PS, photosensitizer; RB, rose bengal; WMH: white metal halide*.

Using MB as a photosensitizer, the concentration required for a bactericidal effect was 0.62 μg/ml at both fluences (18 J/cm^2^ and 37 J/cm^2^) with the 625-nm LED lamp (Figures 4D,E, 5D,E) and at 37 J/cm^2^ with the WMH-lamp (Figures 4F, 5F).

Using RB as a photosensitizer, the concentration required for a bactericidal effect was 0.62 μg/ml for the WMH lamp (Figures 4C, 5C), and 0.31 μg/ml for the 515-nm LED lamp, at fluences of either 18 J/cm^2^ or 37 J/cm^2^ (Figures 4A,B, 5A,B).

### Bactericidal effect of aPDT combined with classical antibiotics

The inhibitory effect of aPDT on *S. aureus* was maintained in the presence of 1 or 10 μg/ml of MU or LN, as evidenced by a 6 log_10_ reduction in all assays. However, by combining aPDT with either of the two antimicrobial agents, the same reduction in bacterial growth was achieved after decreasing photosensitizer concentration by 50%, except in the case of the WMH-light MB-aPDT + 1 μg/ml LN, for which no change was observed (Table 1, Figures 4, 5). The combination of 10 μg/ml MU + RB-aPDT or MB-aPDT using the WMH light allowed for the greatest decrease (>75%) in photosensitizer concentration (≤ 0.03 μg/ml) with respect to the concentration required in the absence of antibiotic (0.62 μg/ml) (Table 1, Figures 4C,F).

In general, using the same antibiotic concentrations and irradiation conditions, an equivalent reduction in bacterial activity was achieved using lower concentrations of RB than of MB. There were 3 exceptions to this observation: 1 μg/ml of LN + WMH light, and both concentrations of MU + WMH light (Table 1).

MU, especially at the higher concentration (10 μg/ml), was more effective than LN in allowing a maximum decrease in the concentration of RB used (0.03 μg/ml), both at the lowest fluence (18 J/cm^2^) with the 515-nm LED light and the highest fluence (37 J/cm^2^) with the 515-nm LED light and theWMH light (Table 1).

### Toxic effects of photosensitizers, antibiotics, and irradiation

At the range of concentrations evaluated and in the same conditions as described for the experiments above, but keeping the samples in darkness, neither photosensitizer reduced the number of CFU/ml in the initial inoculum.

In the absence of photosensitizers and irradiation, the tested concentrations of both antibiotics failed to effectively inactivate the bacteria. A maximum reduction of 0.2 log_10_ was observed for the highest concentration of both antibiotics (10 μg/ml) (Figures 4, 5).

Neither the LED lamp at 18 J/cm^2^ and 37 J/cm^2^ nor the WMH-lamp at 37 J/cm^2^ significantly reduced the number of CFU/ml (reduction of < 0.2 log_10_) (Figures 4, 5).

The cumulative effect of antibiotic alone and irradiation equated to a reduction in the number of CFU/ml of < 0.5 log_10_ (Figures 4, 5).

## Discussion

The present study demonstrates that the combination of MU or LN with MB-aPDT or RB-aPDT exerts a synergistic bactericidal effect against *S. aureus in vitro*. By combining antibiotic with aPDT, the bactericidal effect produced by aPDT alone can be achieved using a much lower photodynamic dose (i.e., lower photosensitizer concentration or lower fluence). Our findings suggest that results obtained with aPDT could be markedly improved by combining this treatment modality with classical antibiotic treatment.

The antimicrobial agents ampicillin, gentamicin, and vancomycin have been previously shown to increase the sensitivity of *Enterococcus faecium* to aPDT, using MB as a photosensitizer (Chibebe Junior et al., 2013). Similarly, gentamicin increases the efficacy of aPDT with 5-ALA against *S. aureus* biofilms (Barra et al., 2015). The combination of vancomycin and aPDT using cationic porphyrins is also highly effective against *S. aureus* biofilms (Provenza et al., 2009). By contrast, Tanaka and coworkers found that both LN and vancomycin decrease the therapeutic effect of MB-aPDT in a murine model of MRSA bacterial arthritis (Tanaka et al., 2013). They hypothesized that in that mouse model aPDT may stimulate antibacterial neutrophil activity, rather than actively killing bacteria, and proposed that LN and vancomycin may inhibit the activation of inflammatory cytokines without eradicating the bacteria, thereby limiting the effect of aPDT. In our study, the combination of LN and MB-aPDT or RB-aPDT resulted in a synergistic bactericidal effect on a *S. aureus* in suspension. To our knowledge, this study is the first to investigate the effect of combining MU antibiotic treatment with aPDT, and demonstrates that the greatest synergistic effect is obtained with MU + aPDT combination, particularly when RB is used as a photosensitizer. Furthermore, to the best of our knowledge this is the first study to investigate the effects of combining antibiotics with aPDT using RB as the photosensitizer.

The effectiveness of RB-aPDT against *S. aureus* (Kato et al., 2012; Nakonechny et al., 2013), including MRSA (Guo et al., 2010), has been previously demonstrated, with green light (Guo et al., 2010) producing a greater reduction in bacterial growth than white light (Kato et al., 2012; Nakonechny et al., 2013). These results are in good agreement with those of the present study. We found that the concentration of RB required to reduce bacterial growth by 6 log_10_ using the WMH light (0.6 ug/ml) was double that required when green light was used (0.3 ug/ml). A list of studies investigating the effect of RB-aPDT on *S.aureus* is provided in Table 2.

**Table 2:** Summary of studies of the *in vitro* efficacy of RB-aPDT on *S.aureus*.

**References**	**Strain**	**Preincubation (min)**	**Intensity (mW/cm^2^)**	**Fluence (J/cm^2^)**	**Emission spectra λ (nm)**	**Concentration RB (μM)**	**Media**	**CFU /ml initial**	**log_10_ reduction**
Nakonechny et al., 2013	ATCC 25923	15–60	1.6	2.88	White	30	PBS	10^7^	1.2
Kato et al., 2012	FDA 209P	ND	25	15	White halogen	1–5	ND	3.10^7^	3.9
Guo et al., 2010	MRSA	3	14	33	525	3	PBS	10^8^	6
Tanaka et al., 2012	MRSA clinical	0	40	5	550	1	HBSS	10^8^	7.5
Present study	ATCC 29213	0	5.8	18–37	515	0.32	Water	10^7^	>6
Present study	ATCC 29213	0	90	37	420–700	0.64	Water	10^7^	>6

*RB, Rose bengal*.

Previous studies have demonstrated the *in vitro* efficacy of MB-aPDT against both *S. aureus* and MRSA (Yow et al., 2011; Kashef et al., 2012; Tanaka et al., 2012) using white light lamps (Zeina et al., 2001; Nakonechny et al., 2013) and red LED lamps (Yow et al., 2011; Huang et al., 2012; Kashef et al., 2012; Vecchio et al., 2015). Although we used higher fluences and lower MB concentrations, our findings are in good agreement with those of previous studies, as shown in Table 3 (Yow et al., 2011; Huang et al., 2012; Nakonechny et al., 2013; Vecchio et al., 2015).

**Table 3:** Summary of studies of the *in vitro* efficacy of MB-aPDT on *S. aureus*.

**References**	**Strain**	**Preincubation (min)**	**Intensity (mW/cm^2^)**	**Fluence (J/cm^2^)**	**Emission spectra λ (nm)**	**Concentration MB (μM)**	**Media**	**CFU/ml initial**	**log_10_ reduction**
Huang et al., 2012	8325-4	30	100	8	660	20	PBS	10^8^	6
Vecchio et al., 2015	NCTC 8325	15	ND	5	660	20	PBS	10^8^	4
Kashef et al., 2012	ATCC 25923	30	91	163.8	660	156.32	PBS	10^4^–10^5^	3.1
Kashef et al., 2012	MRSA	30	91	163.8	660	156.32	PBS	10^4^–10^5^	2.2
Yow et al., 2011	ATCC 25923	0	ND	30	600	3	PBS	10^8^	6.5
Yow et al., 2011	MRSA clinical	0	ND	30	600	3	PBS	10^8^	7
Tanaka et al., 2012	MRSA clinical	0	0.040	20	665	100	HBSS	10^8^	6.5
Nakonechny et al., 2013	ATCC 25923	15	1.6	2.88	White	30	PBS	10^6^	6
Zeina et al., 2001	Oxford	0	42	15.12	400–700	312.65	PBS	10^8^	5.4
Our study	ATCC 29213	0	7	18-37	625	7.6	water	10^7^	6
Our study	ATCC 29213	0	90	37	420–700	7.6	water	10^7^	6

*MB, Methylene blue*.

Selecting a light source with an emission spectrum that corresponds to the absorption spectrum of the photosensitizer should theoretically result in greater efficacy (Calzavara-Pinton et al., 2007). We observed efficient excitation of photosensitizers using either a LED lamp with an appropriate emission λ for each of the 2 photosensitizers tested, or a WHM light that covers the absorption spectra of most photosensitizers, making aPDT easier to perform and avoiding the need to use a specific lamp for each photosensitizer (Soria-Lozano et al., 2015). PDT using artificial white light has been shown to be as effective and well-tolerated as daylight photodynamic therapy (DL-PDT) for actinic keratosis (O'Gorman et al., 2016). DL-PDT is a new PDT modality in which the photosensitizer is activated by sunlight rather than a lamp (Enk et al., 2015; Gilaberte et al., 2015; Morton et al., 2015). The use of daylight makes the PDT procedure simpler and more efficient (de Berker et al., 2007; Wiegell et al., 2012; Vignion-Dewalle et al., 2015). The results of our experiments using a white light lamp suggest that cutaneous infections caused by *S. aureus* could be treated using daylight-activated aPDT combined with either topical (MU) or systemic (LI) antibiotic treatment. However, it should be borne in mind that the present findings were obtained following *in vitro* irradiation of cultured microorganisms, and results could differ when deep tissue penetration of light is required.

To our knowledge, this is the first study to investigate the efficacy of several combinations of PDT and antibiotics in the treatment of *S. aureus*. We found that the most efficacious combination was RB-aPDT using green or white light, the photosensitizer RB, and the antibiotic MU. While we did not examine the effects of this approach *in vivo* or in biofilms, the synergistic effects of aPDT combined with antibiotics described here demonstrate that, at least in a bacterial suspension, the concentration of photosensitizer required to achieve a bactericidal effect is significantly lower than that required with aPDT alone. The main advantage of this combination in terms of clinical application would be a decreased intensity of blue or red staining caused when the photosensitizer is applied to the skin or mucous membranes, making the procedure more cosmetically appealing. Whether this approach would decrease the likelihood of developing antibiotic resistance or overcome existing problems caused by antibiotic-resistant bacteria, as has been proposed (Bartolomeu et al., 2016), remains to be determined.

## Conclusion

The combination of the antibiotics MU or LN with aPDT using the photosensitizers RB or MB results in a synergistic bactericidal effect on *S. aureus in vitro*.

Combining aPDT with concomitant classical antibiotic treatment may produce better results than those obtained using aPDT alone.

## Author contributions

VP participated in the design of the study, conducted all experiments, performed the figures and drafted the manuscript. AR and YG contributed equally to this work: participated in the design of the study, in the analysis and interpretation of data for the work and drafted the manuscript. LP and VL conducted some experiments and helped to draft the manuscript. IG and SB checked our results and have contributed to the interpretation of data for the work and to the resolution of biofilms questions and in the discussion of the final manuscript because they work with us in the project CTQ 2013–48767-C3-2-R from the Spanish Ministry of Science and Innovation studing the effect of photodynamic therapy on biofilms. SN participated in the design of the study and he revised and corrected the manuscript particularly everything related light parameters. MP revised and corrected the manuscript particularly the background and the discussion. All authors read and approved the final manuscript.

### Conflict of interest statement

The authors declare that the research was conducted in the absence of any commercial or financial relationships that could be construed as a potential conflict of interest.
